# Introducing Machine Learning to Detect Personality Faking-Good in a Male Sample: A New Model Based on Minnesota Multiphasic Personality Inventory-2 Restructured Form Scales and Reaction Times

**DOI:** 10.3389/fpsyt.2019.00389

**Published:** 2019-06-06

**Authors:** Cristina Mazza, Merylin Monaro, Graziella Orrù, Franco Burla, Marco Colasanti, Stefano Ferracuti, Paolo Roma

**Affiliations:** ^1^Department of Human Neuroscience, Sapienza University of Rome, Rome, Italy; ^2^Department of General Psychology, University of Padua, Padua, Italy; ^3^Department of Surgical, Medical, Molecular & Critical Area Pathology, University of Pisa, Pisa, Italy

**Keywords:** Minnesota Multiphasic Personality Inventory-2 Restructured Form, faking-good, machine learning, response latency, time pressure

## Abstract

**Background and Purpose.** The use of machine learning (ML) models in the detection of malingering has yielded encouraging results, showing promising accuracy levels. We investigated the possible application of this methodology when trained on behavioral features, such as response time (RT) and time pressure, to identify faking behavior in self-report personality questionnaires. To do so, we reintroduced the article of Roma et al. (2018), which highlighted that RTs and time pressure are useful variables in the detection of faking; we then extended the number of participants and applied an ML analysis.

**Materials and Methods.** The sample was composed of 175 subjects, of whom all were graduates (having completed at least 17 years of instruction), male, and Caucasian. Subjects were randomly assigned to four groups: honest speeded, faking-good speeded, honest unspeeded, and faking-good unspeeded. A software version of the Minnesota Multiphasic Personality Inventory-2 Restructured Form (MMPI-2-RF) was administered.

**Results.** Results indicated that ML algorithms reached very high accuracies (around 95%) in detecting malingerers when subjects are instructed to respond under time pressure. The classifiers’ performance was lower when the subjects responded with no time restriction to the MMPI-2-RF items, with accuracies ranging from 75% to 85%. Further analysis demonstrated that *T*-scores of validity scales are ineffective to detect fakers when participants were not under temporal pressure (accuracies 55–65%), whereas temporal features resulted to be more useful (accuracies 70–75%). By contrast, temporal features and *T*-scores of validity scales are equally effective in detecting fakers when subjects are under time pressure (accuracies higher than 90%).

**Discussion.** To conclude, results demonstrated that ML techniques are extremely valuable and reach high performance in detecting fakers in self-report personality questionnaires over more the traditional psychometric techniques. Validity scales MMPI-2-RF manual criteria are very poor in identifying under-reported profiles. Moreover, temporal measures are useful tools in distinguishing honest from dishonest responders, especially in a no time pressure condition. Indeed, time pressure brings out malingerers in clearer way than does no time pressure condition.

## Introduction

A crucial issue in medico-legal settings is the determination of whether a given symptom presentation or claimed cognitive impairment is credible, particularly when there is an external incentive, such as compensation or secondary gain (1). Recently, an increasing number of studies have addressed the phenomenon of malingering, which refers to an individual’s deliberate choice to distort his/her mental or physical symptoms in order to achieve personal goals or advantages (2–4). More specifically, people can fake a clinical evaluation in two manners: faking-bad or faking-good. Faking-bad involves fabricating or exaggerating symptoms in an attempt to gain secondary benefits (e.g., financial compensation) (5). Faking-good, in contrast, involves presenting oneself in a more positive manner (6). Faking-good behaviors occur with alarming frequency in a variety of settings, from employee selection to forensic evaluation (7), making the prevention and identification of this phenomenon a field of great interest especially for practitioners and also for researchers. For instance, a candidate might lie about his/her personality during an employee selection process in order to secure a job that requires a particular profile. The problem of testing fit to work is crucial when a person is called to cover a position for which certain personality profiles are potentially dangerous; this could apply, for instance, to soldiers, police officers and intelligence staff, train drivers, and pilots. The prevalence of faking-good behaviors is unknown, but it likely exceeds malingering (8). Baer and Miller (9) estimated a dissimulation rate of 30% in job applicants; according to Donovan et al. (10), approximately 50% of applicants admit exaggerating qualities or characteristics of themselves, such as dependability or reliability, and over 60% of applicants de-emphasize their negative attributes. Again, the identification of faking-good subjects is critical in forensic settings, in which individuals can obtain some advantages by presenting themselves favorably (11). This is particularly true in forensic evaluations of parental skills (12) in the context of child custody hearings in which from 20% to as high as 74% of custody litigants (9) are prone to ménage a positive impression of themselves. A similar risk concerns psychological evaluations for obtaining gun or driving licenses. In a study involving offenders referred for impaired driving, Lapham et al. (13) found that about 30% of them lied about substance abuse. Thus, faking-good behavior is an important issue; however, to date, most studies have investigated faking-bad behavior [for a review, see Ref. (14)], and faking-good behavior remains underinvestigated (15).

The literature shows that faking is difficult to detect on the basis of a clinical interview only (16–18). For this reason, psychometric techniques have been proposed to evaluate systematic distortions concerning psychiatric symptoms. Validity scales of personality questionnaires are traditionally the main measure used to detect fakers by assessing the presence of responding biases (19) (i.e., the systematic tendency to answer items on a personality inventory in a manner inconsistent with accurate self-presentation). The validity scales of the Minnesota Multiphasic Personality Inventory (MMPI) (20, 21), also in its restructured form (MMPI-2-RF) (22), are the most suitable prototypes for this purpose. These scales allow researchers to measure response consistency, the presence of overexaggerating symptoms (23), and symptom minimization (underreporting) patterns (24). Specifically, the logic behind the underreporting Lie scale (L-r) and the Correction-Defensiveness scale (K-r) is that only people who want to provide a socially virtuous and well-adapted image of themselves will not answer genuinely those items that refer to common behaviors or small infractions that the majority of individuals are keen to admit to (e.g., “Sometimes I get angry”). However, validity scales are not always effective for detecting faking, as many items show high transparency; thus, test takers are often able to discern the constructs that the items are designed to measure and feign their answers towards the desired direction.

On the basis of this observation, many authors have searched for indirect measures of simulation. In 1972, for example, Dunn and colleagues (25) suggested that response times (RTs) to single items on a personality questionnaire could be used to distinguish malingerers from honest respondents, considering that the cognitive processes involved in lying are different from those involved when a person answers truthfully. As lying is a more complex mental operation than honesty, and because of the additional cognitive processing that is assumed to be involved with faking, simulators are exposed to a greater cognitive load than are truth tellers. Consequently, simulators are expected to obtain longer RTs than are controls and truth tellers (26, 27). A recent meta-analysis (6) on the relationship between RT and faking confirmed that honest respondents take less time to answer. The difference observed in RTs between faking and honest respondents is statistically significant only when test takers endorse items; it is not present when items are rejected. Similar evidence has been produced by researchers investigating the behavioral responses of honest and faking subjects using more complex measures, such as mouse tracking (28, 29) and keystroke dynamics (30, 31). Moreover, time pressure is a technique that has been shown to be effective in identifying malingering respondents (32). Research has shown that speeded tests, which impose time constraints by asking test takers to answer as quickly as possible, may increase accuracy in detecting fakers. In this context, Sutherland and Spilka (33) reported that time pressure accentuated a response style oriented to social desirability. Khorramdel and Kubinger (3) reported that the effect of time pressure on accentuating faking-good behavior is greater with a dichotomous response format. The rationale behind this phenomenon is that malingerers under time constraint pay less attention to the item selection and endorse more socially desirable items than they normally would, generating less believable profiles.

Roma et al. (34, in press) recently conducted a study of the faking-good personality profile, measuring RTs in a time pressure/no time pressure condition. In their experimental paradigm, participants were randomly assigned to one of four groups, each with different instructions based on the two manipulated factors (honest vs. faking-good; speeded vs. unspeeded). Interestingly, the authors found significant differences in terms of test fulfillment time and L-r/K-r scale completion time in both the time pressure and no time pressure conditions. The speeded condition increased *T*-scores in the L-r and K-r scales but decreased *T*-scores in some of the Restructured Clinical (RC) scales.

More recently, lie detection research machine learning (ML) models, which comprise “a category of algorithms that allow software applications to become more accurate in predicting outcomes without being explicitly programmed,” have been used to distinguish between faking and honest respondents in many contexts, from the detection of false identities (35) to the detection of faked depression (5), with extremely promising accuracy levels. In the latter study, for instance, ML models were trained on behavioral features (e.g., number of symptoms of depression declared, mouse trajectory, and RTs) collected from depressed patients and malingerers; the resulting algorithms correctly identified malingerers with an accuracy approaching 96%. Indeed, ML has been demonstrated to outperform traditional statistical methods in terms of model complexity and classification accuracy in a wide variety of fields, including neuroimaging (36).

Here, we extend the results reported by Roma et al. (1) investigating whether the adoption of ML techniques may improve the detection of faking-good behavior, relative to traditional psychometric techniques.

## Materials and Methods

### Participants and Research Design

Roma et al. (1) initially collected 140 young adult volunteers over a period of 2 months, from October to November 2017. These subjects participated in the study for a small reward (European breakfast in a café). To limit confounding variables, all subjects were aged 25 to 30 years (*M* = 26.64, *SD* = 1.88 years), healthy male (i.e., male without a diagnosed psychiatric disorder), Caucasian, and (non-psychology) graduates having completed at least 17 years of education. Participants were randomly assigned to one of four research groups, defined by a combination of the two manipulated factors relating to instruction (honest vs. faking-good) and time pressure (speeded vs. unspeeded): honest without time pressure (*n* = 33), faking-good without time pressure (*n* = 34), honest speeded (*n* = 35), and faking-good speeded (*n* = 33). In the unspeeded condition, participants were instructed to take all the time they needed to choose their answer, whereas in the speeded condition, participants were asked to answer as fast as they could, but no actual time limitation was imposed on them. Five subjects were excluded from data analysis for one or more of the following reasons: (a) failure to follow instructions as assessed by the final request (*n* = 2), (b) one or more changes in answers (*n* = 2), or (c) too brief a latency in one or more responses (*n* = 1, 3,000 ms). The final sample was composed of 135 subjects. No statistically significant differences were observed on age or level of education between groups.

Subsequently, from September to October 2018, we recruited an additional 45 young adult volunteers, whom we intended to use as an out-of-sample evaluation group for the models, built on the original sample collected by Roma et al. (1). All participants were rewarded with a breakfast ticket. Subjects were all tested in the morning and were randomly assigned to one of the four instruction groups listed above. Five subjects were excluded from the data analysis for one of the following reasons: (a) failure to follow instructions as assessed by the final request (*n* = 3) or (b) too brief a latency in one or more responses (*n* = 2, 3,000 ms). The remaining 40 persons were aged 23 to 32 years (*M* = 27.10, *SD* = 2.24 years), male, Caucasian, and (non-psychology) graduates. No statistically significant differences were observed on age or level of education. Overall, 175 young adult volunteers were recruited. Our samples were composed only of males both in an attempt to limit confounding variables and because researches indicated that men are more likely than women to use form of deception such as lying to obtain what they want (37) and to engage in harsher form of impression management (38, 39). Moreover, according to Volkema (40), women maintain higher ethical standards than do men. Such findings have been recently confirmed by Hogue et al. (41), which show that men have greater intentions than women to invent untrue personal information.

### Experimental Procedure and Stimuli

The experimental procedure and stimuli were the same as those used and described in the research conducted by Roma et al. (34). In more detail, after filling in the demographic questionnaire and reading the instructions for the research task relative to their assigned group, participants responded to MMPI-2-RF items that were loaded onto the Microsoft Excel platform. Finally, their understanding of the instructions was checked. For more details on the materials and methods, please refer to Roma et al. (34). For each participant, 21 independent variables were collected. These independent variables included latencies (temporal features) and raw scores for each of the MMPI-2-RF scales (see Table 1).

**Table 1:** Features calculated for each participant.

	Feature	Description
**Temporal performance**	Total time (tt)	Time taken to compile the entire Minnesota Multiphasic Personality Inventory-2 Restructured Form (MMPI-2-RF) protocol
	1st part time (1t)	Time taken to compile the first part (items 1–112) of the MMPI-2-RF protocol
	2nd part time (2t)	Time taken to compile the second part (items 113–224) of the MMPI-2-RF protocol
	3rd part time (3t)	Time taken to compile the third part (items 225–338) of the MMPI-2-RF protocol
	L-r time (Lrt)	Time taken to respond to the L-r scale items
	K-r time (Krt)	Time taken to respond to the K-r scale items
	F-r time (Frt)	Time taken to respond to the F-r scale items
	Neutral time (Nt)	Time taken to respond to the 10 neutral questions
***T*** **-score**	L-r *T*-score (L-r)	*T*-score obtained in the L-r scale
	K-r *T*-score (K-r)	*T*-score obtained in the K-r scale
	F-r *T*-score (F-r)	*T*-score obtained in the F-r scale
	RCd *T*-score (RCd), RC1 *T*-score (RC1), RC2 *T*-score (RC2), RC3 *T*-score (RC3), RC4 *T*-score (RC4), RC6 *T*-score (RC6), RC7 *T*-score (RC7), RC8 *T*-score (RC8), RC9 *T*-score (RC9)	*T*-scores obtained in the RCd, RC1, RC2, RC3, RC4, RC6, RC7, RC8, and RC9 scales, respectively
	Total RC *T*-score (RCtot)	Sum of the *T*-scores obtained in all MMPI-2-RF scales

### Machine Learning Models: General Method

We performed two ML analyses: the first aimed at classifying participants under time pressure and the second aimed at classifying participants without time pressure. Analyses were run in WEKA 3.9 (42) following a best practice workflow: feature selection, model training, and then model testing in an out-of-sample group (41). Following standard practice, given the high number of independent variables, the optimal subset was used in model building. Features selection is a widely used procedure in the construction of ML models (44), aimed at removing redundant and irrelevant features in order to increase the model generalization by reducing overfitting (45) and noise in the data. In this experiment, non-redundant features were extracted on the basis of their correlation with the outcome (faking vs. non-faking) and their mutual intercorrelation. In other words, we singled out the features that were more correlated with the predicting classification (faking vs. honest) and less correlated with one another. This procedure was performed using a correlation-based feature selector (CFS) (44), as implemented in WEKA 3.9 (42). The CFS algorithm, using a “greedy stepwise” search method, evaluates the worth of a subset of features by considering the individual predictive ability of each feature along with the degree of redundancy with other predictors. Subsets of features that were highly correlated with the classification (the dependent variable) but with low intercorrelation were selected. For each selected predictor, we reported the point biserial correlation coefficient (*r*_pb_), which related to the correlation with the outcome variable, and the correlation matrix with the other selected features.

The predictors resulting from the feature selection were fed as inputs to a number of ML models in order to evaluate the accuracy of the subjects’ classification as faking or honest. Models were trained on the first sample of participants collected by Roma et al. (34), called the training set, following a 10-fold cross-validation procedure (46). K-fold cross-validation is a technique used to evaluate predictive models by repeatedly partitioning the original sample (e.g., 60 participants) into a training set to train the model, and a validation set to evaluate it. Specifically, in 10-fold cross-validation, the original sample is randomly partitioned into 10 equal-size sub-samples, or folds (e.g., 10 sub-samples of 6 participants each). Of the 10 sub-samples, a single sub-sample is retained as validation data to test the model, and the remaining 9 sub-samples are used as training data. The process is repeated 10 times, with each of the 10 folds used exactly once as validation data. The results from the 10 folds are then averaged to produce a single estimation of prediction accuracy.

In order to evaluate the model’s capacity for generalization, it was tested on completely new data to reduce bias (47). Because classifiers are built to fit the data, it is important to know how an existing model fits unseen data. For this reason, we collected a new group of participants to evaluate the real performance of the classifiers. Data were collected by a different experimenter, and subjects were randomly assigned to the experimental conditions, in order to eliminate *a priori* knowledge of how the classifiers work during the test collection. The sample size of the test group was 40 subjects, corresponding to approximately 30% of the training sample—a percentage that is usually regarded as satisfactory (48). For each model, we reported accuracy, recall (sensitivity or true positive rate), and precision.

As stated above, we evaluated the accuracy of different ML classifiers in order to investigate whether the results were stable across classifiers and independent of specific model assumptions. In fact, the algorithms that we chose were representative of different underlying classification strategies, as follows:

- Logistic regression: measures the relationship between the categorical dependent variable and the independent variables by estimating probabilities using a logistic function (49).
- Support vector machine (SVM): a binary linear classifier that maps the space and divides the examples of separate categories by as large a margin as possible (50, 51).
- Naive Bayes: a probabilistic classifier based on Bayes’ theorem, which assumes independence between features (52).
- Random forest: an ensemble learning method that operates by constructing a multitude of decision trees and combining their results (53).
- Logistic model tree (LMT): combines logistic regression and decision tree learning (54).

ML models, such as some of those reported above, are difficult to interpret. Often, the mechanics that yield the algorithm to identify a single participant as honest or faking-good is unclear. For this reason, ML models are sometimes analyzed on the basis of decision rules such as a tree model called J48 (55). This is one of the simplest—if not the simplest—classifier in terms of the transparency of operations, and it highlights the classification logic (albeit not in the most efficient way) (56). In our research, it was helpful to use this method to explain the operations performed by the algorithm.

All algorithms were run using default parameters set by WEKA 3.9 (41). Therefore, there was no fine-tuning of the parameters to increase classification accuracy.

## Results

### No Time Pressure Models

Sixty-seven participants (33 honest and 34 faking) from Roma et al. (34) were used to train the models, whereas the 40 new participants (10 honest and 10 faking) collected for this study were used to test the model. All participants completed the MMPI-2-RF without time pressure.

**Figure 1:**
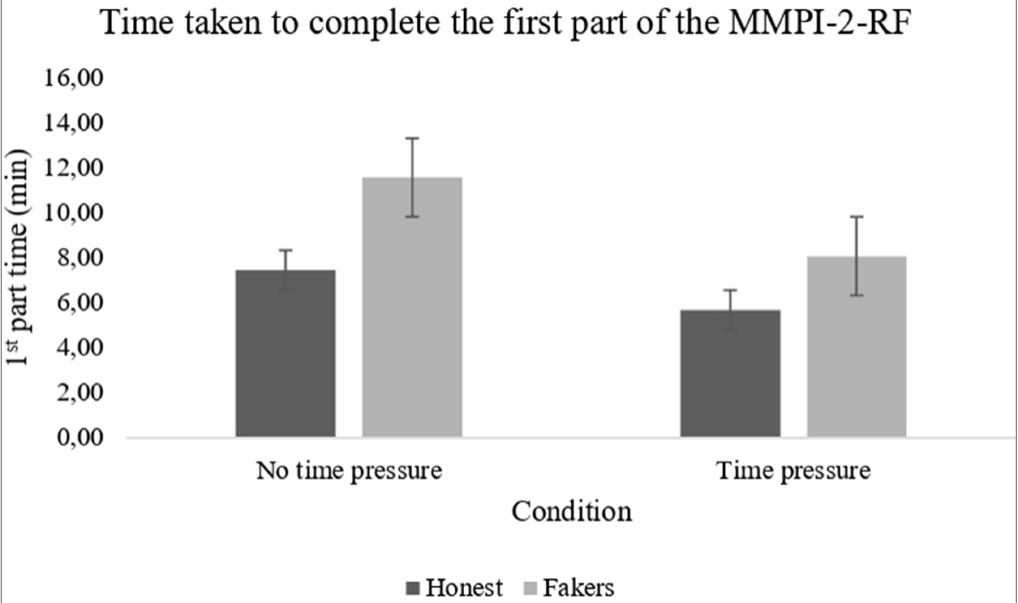
The bar plots represent the time taken by participants in different experimental conditions to complete the first part of the MMPI-2-RF protocol.

The feature selection, which was run as described above, identified the following predictors: first part time (1t), K-r time (Krt), RC4 *T*-score (RC4), and RC9 *T*-score (RC9). Table 2 reports the correlation matrix between each selected feature and the outcome variable (faking vs. non-faking). The time taken by the subject to complete the first part of the MMPI-2-RF turned out to be the feature that best distinguished the two groups, as faking-good respondents were, on average, slower than honest respondents in responding to the first 112 MMPI-2-RF items (faking *M* = 11.59 min, *SD* = 1.28; honest *M* = 7.46 min, *SD* = 0.99; see **Figure 1**). Moreover, it is worth noting that the MMPI-2-RF validity scales (L-r, F-r, and K-r) did not contribute to the identification of faking behavior.

**Table 2:** The table reports the correlation matrix for the four features selected by the CFS algorithm in the group of participants under time pressure. The point biserial correlation (*r*_pb_) between each selected feature and the dependent variable (faking vs. honest) is also reported.

	1t	Krt	RC4	RC9	Faking vs. honest
1t	1.00	0.31	−0.27	−0.15	0.88
Krt	0.31	1.00	−0.28	−0.42	0.42
RC4	−0.27	−0.28	1.00	0.02	−0.36
RC9	−0.15	−0.42	0.02	1.00	−0.31
Faking vs. honest	0.88	0.42	−0.36	−0.31	1.00

**Figure 2:**
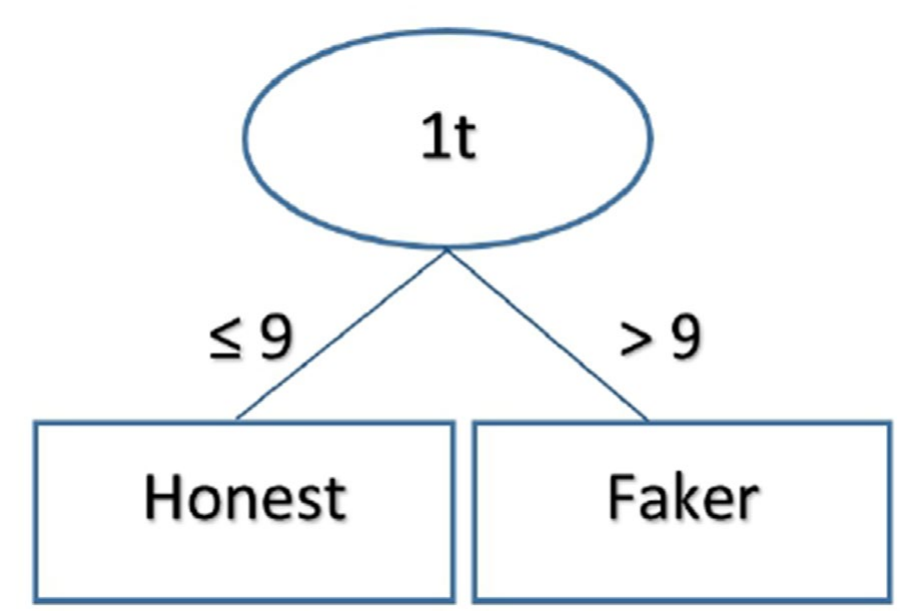
The figure reports the rules that the J48 decision tree used to classify participants as faking-good or honest in the no time pressure sample. According to this algorithm, subjects who took fewer than 9 minutes to complete the first part of the questionnaire were classified as honest, whereas subjects who took more than 9 minutes were classified as faking.

The results obtained by different ML algorithms in the training set and the test set are reported in **Table 3**. It is noticeable that all classifiers reached a very high accuracy (97–100%) in the training set. However, the accuracy in the test set dropped to 75%, with the logistic classifier outperforming other classifiers (logistic accuracy = 85%). These results indicate that out-of-sample accuracy was degraded, despite the errors being equally distributed amongst faking-good and faking-bad behavior. In **Figure 2**, the output of a J48 tree (used to facilitate understanding of a classification strategy) is reported. The algorithm achieved an accuracy of 95.9% (recall = 0.956, precision = 0.956) in the training set and 75% in the test set (recall = 0.750, precision = 0.753). It should be noted that J48 bases its outcome exclusively on the time spent by each participant in completing the first part of the MMPI-2-RF.

**Table 3:** The table reports the accuracy, recall, and precision measures for each ML model. Results are reported for the 10-fold cross-validation (training) set and the test set, for both the time pressure and no time pressure groups.

	Training set (10-fold cross-validation)			**Test set**		
	Accuracy	Recall	Precision	Accuracy	Recall	Precision
*No time pressure models*						
Logistic	100%	1.00	1.00	85%	0.85	0.854
SVM	98.53%	0.985	0.986	75%	0.750	0.753
Naive Bayes	100%	1.00	1.00	75%	0.750	0.753
Random forest	98.53%	0.985	0.986	75%	0.750	0.753
LMT	97.06%	0.971	0.972	75%	0.750	0.775
*Time pressure models*						
Logistic	98.51%	1.00	0.986	95%	0.95	0.955
SVM	98.51%	0.985	0.986	95%	0.95	0.955
Naive Bayes	100%	1.00	1.00	95%	0.95	0.955
Random forest	97.01%	0.970	0.970	95%	0.95	0.955
LMT	95.52%	0.955	0.959	95%	0.95	0.955

### Time Pressure Models

Sixty-eight participants (35 honest and 33 faking) from Roma et al. (34) were used to train the models, whereas 20 new participants (10 honest and 10 faking) were used for out-of-sample testing. All participants performed the MMPI-2-RF under time pressure.

The CFS feature selector identified the following predictors: first part time (1t), third part time (3t), total time (tt), L-r time (Lrt), K-r time (Krt), L-r *T*-score (L-r), F-r *T*-score (F-r), and RC4 *T*-score (RC4). **Table 4** reports the correlation matrix between each feature and the dependent variable (faking vs. honest). Also, in this case, the time used to complete the first part of the MMPI-2-RF protocol was the variable that best discriminated between the two samples (faking vs. honest), with faking-good respondents taking longer than honest respondents (faking *M* = 8.09 min, *SD* = 1.25; honest *M* = 5.69 min, *SD* = 1.06).

**Table 4:** The table reports the correlation matrix for the eight features selected by the CFS algorithm in the no time pressure group. The point biserial correlation (*r*_pb_) between each selected feature and the dependent variable (faking vs. honest) is also reported.

	1t	3t	tt	Lrt	Krt	L-r	F-r	RC4	Faking vs. honest
1t	1.00	0.05	0.72	0.67	0.68	0.67	−0.17	−0.27	0.72
3t	0.05	1.00	0.65	0.35	0.36	0.32	−0.14	−0.22	0.37
tt	0.72	0.65	1.00	0.74	0.77	0.73	−0.16	−0.33	0.83
Lrt	0.67	0.35	0.74	1.00	0.86	0.75	−0.15	−0.29	0.84
Krt	0.68	0.36	0.77	0.86	1.00	0.81	−0.22	−0.37	0.88
L-r	0.67	0.32	0.73	0.75	0.81	1.00	0.03	−0.33	0.83
F-r	−0.17	−0.14	−0.16	−0.15	−0.22	0.03	1.00	0.08	−0.28
RC4	−0.27	−0.22	−0.33	−0.29	−0.37	−0.33	0.08	1.00	−0.37
Faking vs. honest	0.72	0.37	0.83	0.84	0.88	0.83	−0.28	−0.37	1.00

**Table 3** reports the results obtained by different ML algorithms in the 10-fold cross-validation and the test set. All ML models reached 95–100% accuracy in the training set, and similar results were achieved in the test set (95% for all classifiers). In this case, the trained classifiers showed good generalization when tested on a completely new sample. Errors were equally distributed across the two classes, with a similar rate of faking-good and faking-bad behavior.

**Figure 3:**
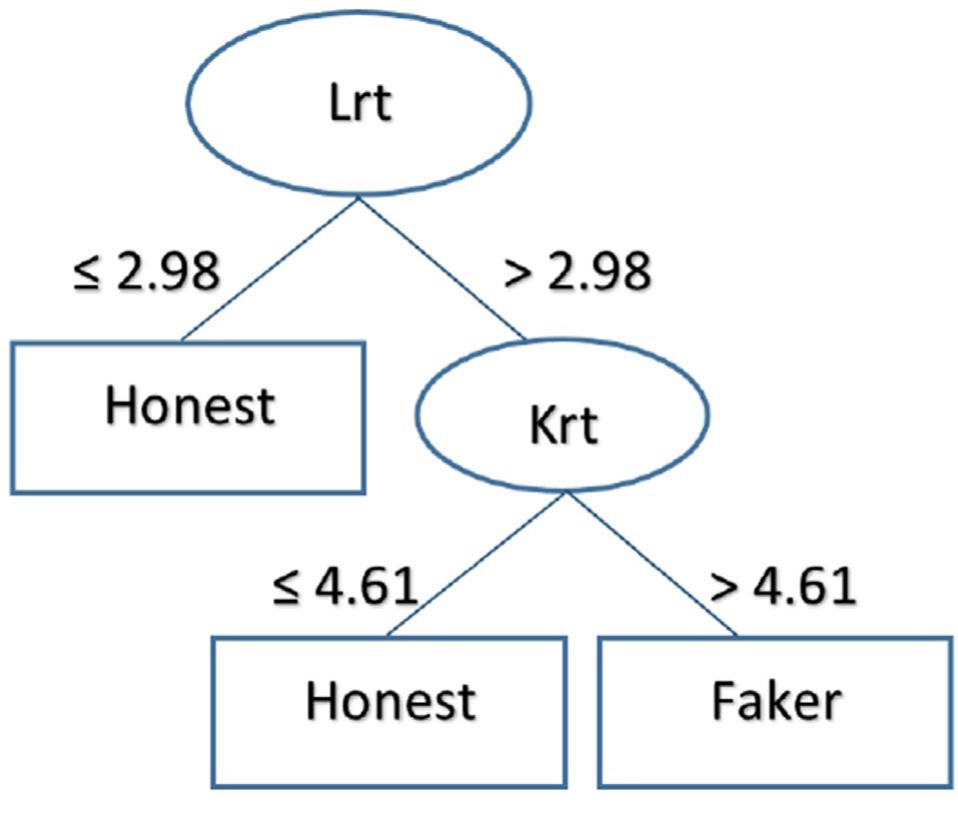
The figure represents the classification logic of the J48 decision tree for the group under temporal pressure. According to the tree, subject who took fewer than 2.98 minutes to fill in the items of the L-r scale were classified as honest; subjects who took more than 2.98 minutes were classified as faking. Subjects who took fewer than 4.61 minutes to complete the K-r scale items were classified as honest; subjects who took more than 4.61 minutes were classified as faking.

Finally, **Figure 3** describes the output of the J48 algorithm. To classify subjects as honest or faking, the classification rule considers the time used to respond to L-r scale items, followed by K-r scale items. The algorithm achieved an accuracy of 92.53% (recall = 0.925, precision = 0.926) in the training set, which remained stable in the test set (accuracy = 90%, recall = 0.900, precision = 0.917). Again, temporal features were sufficient to identify faking responders.

### Models Based Only on the MMPI-2-RF Validity Scales

As reported above, in the no time pressure condition, the MMPI-2-RF validity scales were not included as predictors. This means that the time required to respond to the questionnaire may be sufficient to detect faking-good respondents with a level of accuracy that is greater than chance (75%). The same was shown by the J48 model built on time pressure data, which reached an accuracy of 90% based only on temporal predictors (Lrt and Krt).

In order to address the question “How useful are temporal features compared to traditional validity scales in the identification of faking-good respondents?,” (i) we applied the MMPI-2-RF suggested criteria (see 22) to identify tests showing clear underreporting, and then (ii) we ran new ML models using only the *T*-scores of the validity scales (L-r, F-r, and K-r) as input. Similarly, we ran classifiers using only temporal features (tt, 1t, 2t, 3t, Lrt, Frt, Krt, and Nt) as input.

According to the MMPI-2-RF manual (22), a linear *T*-point score ≥ 80 in the L-r scale is a valid and reliable cut-off for identifying underreporting, as well as a *T*-score ≥ 70 in the K-r scale. Based on these suggested cut-offs, in the original sample of Roma et al. (34), only 12 out of 135 MMPI-2-RF protocols were surely invalid due to underreporting, generating an accuracy in detecting faking-good respondents of only 8.8%. Applying the same criteria to the 40 subjects in the validation study, we did not identify any invalid MMPI-2-RF protocols due to underreporting; that is, we did not detect any faking respondents, in either the time pressure or the no time pressure condition.

Results from new ML models using only *T*-scores of the validity scales or temporal features as input are reported in **Table 5**, for both time pressure and no time pressure conditions. With respect to the no time pressure condition, the *T*-scores of the validity scales were very poor in detecting faking-good behavior. Indeed, model accuracies ranged from 55% to 65%, just above chance. Considering only temporal features, model performance improved slightly (10–15%), reaching an accuracy of 70–75%. In regard to the temporal pressure condition, both validity scale scores and temporal features were good predictors of faking behavior when time pressure instructions were given. In this scenario, all models achieved greater than 90% accuracy.

**Table 5:** The table reports the results of the ML models using only *T*-scores of the validity scales (L-r, F-r, and K-r) as input. Results for the ML models using only temporal features (tt, 1t, 2t, 3t, Lrt, Frt, Krt, and Nt) as input are also reported. Results refer to accuracy, recall, and precision.

	Models based only on T-scores of the validity scales			Models based only on temporal features		
	Accuracy	Recall	Precision	Accuracy	Recall	Precision
*No time pressure models*						
Logistic	60%	0.600	0.600	75%	0.750	0.753
SVM	60%	0.600	0.604	70%	0.700	0.738
Naive Bayes	55%	0.550	0.551	75%	0.750	0.753
Random forest	65%	0.650	0.700	70%	0.700	0.708
LMT	55%	0.550	0.551	75%	0.750	0.775
J48	55%	0.550	0.551	75%	0.750	0.753
*Time pressure models*						
Logistic	95%	0.95	0.955	90%	0.900	0.900
SVM	95%	0.95	0.955	95%	0.950	0.955
Naive Bayes	90%	0.900	0.900	95%	0.950	0.955
Random forest	95%	0.95	0.955	95%	0.950	0.955
LMT	95%	0.95	0.955	95%	0.950	0.955
J48	90%	0.900	0.917	90%	0.900	0.917

## Discussion

Most cognitive and behavioral symptoms can be easily faked, even by naive, non-coached examinees; for this reason, psychometric tools are needed to objectively confirm whether test scores accurately reflect dysfunctions or whether respondents have attempted to simulate or overexaggerate difficulties (57). While malingering is a widely studied topic, there is a lack of research on methods and strategies to detect faking-good behavior (34, 58, 59). Most investigations have focused on techniques to spot faking-bad, rather than faking-good, behavior. However, in many legal conditions (e.g., child custody hearings), examinees are prone to faking-good. Recent advances in psychometric tools have indicated that ML techniques may boost classification accuracy, relative to standard statistical techniques. Accordingly, the goal of this research was to apply ML analysis in the identification of faking-good MMPI-2-RF test takers.

The results showed that ML algorithms achieved very high accuracy in detecting fakers when subjects were instructed to respond under time pressure (in fact, in the out-of-sample test set, all trained models showed an accuracy of 95%). However, the performance of classifiers was lower when subjects responded without time restriction to the MMPI-2-RF items, with accuracies ranging from 75% to 85% in the test set.

To demonstrate whether ML analysis can detect fakers more accurately than traditional validity scales, we detected invalid protocols for underreporting following the MMPI-2-RF suggested criteria. Using these criteria on the very same set of participants that we used to compute the algorithms accuracy resulted in no identification.

Moreover, to investigate whether validity scales are useful for the detection of faking behavior, we ran two sets of ML models: one using only the *T*-scores of the validity scales (L-r, F-r, and K-r) as features and the other using only temporal features (tt, 1t, 2t, 3t, Lrt, Frt, Krt, and Nt). The results showed that the *T*-scores of the validity scales were ineffective for detecting fakers when participants were not under time pressure (achieving only 55–65% accuracy), whereas temporal features were more useful (achieving 70–75% accuracy). By contrast, temporal features and the *T*-scores of the validity scales were equally effective in detecting faking behavior when subjects were under time pressure (achieving accuracies > 90%). Results indicate that time pressure increase faking-good respondents’ descriptions of socially desirable behavior. This result is consistent with previous literature (3, 34, 60, 61) that show that time pressure prevents subjects to think deeply about the content of the questions and the possible lack of credibility of their responses. In other words, time limitations urge people to focus on responding faster, and this accentuates their fake behavior and prevents them from taking the time to consider whether their responses are exaggeratedly good, thus breaking the warning instruction (“your deception should not be detected”).

To conclude, the results suggest that time—in the form of both RTs and time pressure—is a critical factor in the detection of faking behavior. Moreover, the use of ML is extremely valuable and offers the following advantages: first, it detects faking-good respondents on the MMPI-2-RF with significantly higher accuracy than do the validity scales criteria published in the manual; second, it works automatically, so it is more objective than human evaluation; third, it considers a variety of parameters, making it nearly impossible for fakers to successfully cheat; and finally, its predictions can be applied to completely new subjects, strengthening the replicability of the results. It can therefore be concluded that i) the MMPI-2-RF manual criteria with respect to the validity scales are very poor in identifying underreporting and ii) temporal measures are useful for distinguishing between honest and faking respondents, especially in a no time pressure condition.

Widely, our results found that time pressure revealed fakers more clearly than did a no time pressure condition. The ML models in the former condition were also more generalizable. It is reasonable, therefore, to conclude that time pressure, which forces subjects to respond to a self-report questionnaire as quickly as possible, can effectively facilitate the detection of simulators. When it is not possible to instruct participants to respond with maximum speed, as is usually the case in forensic settings, the validity scales of the MMPI-2-RF are insufficient to accurately detect fakers; therefore, it is important to record RTs. To summarize, time pressure is the most reliable method to identify faking-good behavior. However, in the absence of time pressure, RTs are a more accurate measure than validity scales.

Despite that faking-good remains underinvestigated (15), it is a widespread behavior that commonly occurs in all that settings in which individuals are prone to ménage a positive impression of themselves. In employee selection, for instance, 30% of the candidates tend to provide an improved and socially adapted self-image in order to gain a job position. In forensic setting, furthermore, from 20% to as high as 74% of child custody litigants tend to deny or omit negative features of their personality in order to present themselves in a better light, to show more adaptive psychological and behavioral functioning, and to appear as responsible caregivers who will provide for the best interests of their child. A similar risk concerns psychological evaluations for obtaining gun or driving licenses. The present study adds useful insight to the debate over the methods that can be effectively used to detect faking-good behaviors. Based on findings described herein, personality assessment in personnel and forensic contexts could be improved, for example, by introducing time pressure asking subjects to fulfill self-report questionnaires (e.g., MMPI-2-RF) as soon as possible or again, using software that could record the reaction times to test item. To the best of our knowledge, this study was the first to have applied ML to bring out good-fakers.

## Strengths and Limitations

The present study meant to overcome one of the limitations of the previous research conducted by Roma et al. (34) by expanding the sample size. At the same time, it also provides insight into the use of ML models for the detection of faking behavior. The main limitation of the study, however, is that the sample was selected for specificity (graduate males aged 23–32 years), and this reduces the generalizability of the findings. One important future direction would be to test the accuracy of the ML algorithms developed in this study on the forensic population. Future research could also analyze whether limiting the time available to fulfill a self-report personality questionnaire (rather than simply imposing time pressure) could lead to the same results, as such an approach could more easily be employed in forensic settings and personnel selection.

## Data Availability Statement

The dataset used and analyzed in the current study is available from the corresponding author upon reasonable request.

## Ethics Statement

This study was carried out with written informed consent by all subjects, in accordance with the Declaration of Helsinki. It was approved by the local ethics committee (Board of the Department of Human Neuroscience, Faculty of Medicine and Dentistry, Sapienza University of Rome).

## Author Contributions

PR, SF and FB conceived the experiment. Data acquisition was done by PR, CM and MC. Data analysis was done by MM and CM. Data interpretation was done by MM, CM, PR, and GO. Drafting of the manuscript was done by MM, CM, PR, and GO. All authors revised the manuscript critically and gave final approval of the version to be published.

## Conflict of Interest Statement

The authors declare that the research was conducted in the absence of any commercial or financial relationships that could be construed as a potential conflict of interest.

The handling Editor declared a shared affiliation, though no other collaboration, with one of the authors MM.
